# Effect of Trehalose and Lactose Treatments on the Freeze-Drying Resistance of Lactic Acid Bacteria in High-Density Culture

**DOI:** 10.3390/microorganisms11010048

**Published:** 2022-12-23

**Authors:** Shumao Cui, Mengyu Hu, Yuanyuan Sun, Bingyong Mao, Qiuxiang Zhang, Jianxin Zhao, Xin Tang, Hao Zhang

**Affiliations:** 1 State Key Laboratory of Food Science and Technology, Jiangnan University, Wuxi 214122, China; 2 School of Food Science and Technology, Jiangnan University, Wuxi 214122, China; 3 National Engineering Research Center for Functional Food, Jiangnan University, Wuxi 214122, China

**Keywords:** disaccharide, lactic acid bacteria, high density, freeze-drying, survival

## Abstract

Freeze-drying is a commonly used method in commercial preparations of lactic acid bacteria. However, some bacteria are killed during the freeze-drying process. To overcome this, trehalose and lactose are often used as protective agents. Moreover, high-density culture is an efficient way to grow bacterial strains but creates a hypertonic growth environment. We evaluated the effects of trehalose and lactose, as a primary carbon source or as an additive in fermentation, on the freeze-drying survival of *Lactobacillus fermentum* FXJCJ6-1, *Lactobacillus brevis* 173-1-2, and *Lactobacillus reuteri* CCFM1040. Our results showed that *L. fermentum* FXJCJ6-1 accumulated but did not use intracellular trehalose in a hypertonic environment, which enhanced its freeze-drying resistance. Furthermore, genes that could transport trehalose were identified in this bacterium. In addition, both the lactose addition and lactose culture improved the freeze-drying survival of the bacterium. Further studies revealed that the added lactose might exert its protective effect by attaching to the cell surface, whereas lactose culture acted by reducing extracellular polysaccharide production and promoting the binding of the protectant to the cell membrane. The different mechanisms of lactose and trehalose in enhancing the freeze-drying resistance of bacteria identified in this study will help to elucidate the anti-freeze-drying mechanisms of other sugars in subsequent investigations.

## 1. Introduction

Lactic acid bacteria are a group of microorganisms that ferment carbohydrates to produce large amounts of lactic acid [1], and they have excellent probiotic effects, such as regulating microbiota balance, enhancing immunity, and alleviating allergies [2,3]. *Lactobacillus* preparations are lyophilized products prepared by vacuum freeze-drying in the presence of freeze-drying protectants. After freezing and drying, both of which are extreme processes, the bacteria are subjected to mechanical damage, solute damage, cell membrane damage, protein denaturation and inactivation, and DNA damage, which greatly reduce their survival rate [4]. Therefore, improving the survival rate of lyophilized lactic acid bacteria is a major challenge in the commercial production of lactic acid bacteria.

A common method for improving freeze-drying survival is the addition of freeze-drying protectants prior to freeze-drying, with sugars being the most common type of protectant. There are multiple possible mechanisms behind the anti-freeze-drying effect of sugars. The water replacement theory suggests that sugars displace water molecules and thus stabilize the structure of phospholipid cell membranes during drying. The glass transition theory states that sugars can lodge between the cell wall and the cell membrane and increase the glass transition temperature, thus mitigating mechanical damage due to the ice crystal formation in the bacterium [4]. Therefore, sugars such as trehalose, lactose, sucrose, and oligoisomaltose are considered excellent freeze-drying protectants. In particular, trehalose and lactose are the most widely studied freeze-drying protectants. Trehalose is a non-reducing disaccharide that has been widely studied for use as a compatible solute and is extremely useful for the long-term preservation of plant crops and microorganisms and is known for resistance to environmental stresses such as low temperature, dryness, and high salinity [5]. In a previous study, Sun et al. prepared whey protein-pullulan polysaccharide hydrogels as a wall material for microcapsules by adding trehalose, which effectively improved the viability of *Lactobacillus plantarum* during freeze-drying and storage [6]. A combination of trehalose, sucrose, and skimmed milk was shown to be the most effective protectant, leading to survival rates of between 83% and 85% after freeze-drying [7]. In addition, trehalose has been considered a “chaperone molecule” that plays an important role in stabilizing cell membranes under drying and heat stresses [8]. However, sugars, as freeze-drying protectants, are added in large quantities before the bacterium is freeze-dried, leading to waste and expensive production. Therefore, examining other mechanisms of the anti-freeze-drying ability of sugars is necessary. 

Owing to the diverse mechanisms available to alleviate freeze-drying damage, there have been various studies on the addition of sugars, including extracellular polysaccharides, intracellular compatible solutes, intermembrane protectants, and the use of co-envelope materials to improve the freeze-drying survival of bacteria. The presence of carbohydrates on both sides of the bacterial membrane protects not only the bacterial membrane but also cytoplasmic proteins [9]. The presence of small amounts of extracellular polysaccharides may enhance the resistance of a bacterium to freeze-drying; it has been demonstrated that a bacterium undergoes different extracellular polysaccharide production under culture with a specific carbon source, thus improving freeze-drying survival [10]. Numerous studies have found that some organisms and bacteria under stress from heat, cold, acid, high osmosis, substrate deficiency, and oxidation develop adaptive mechanisms, such as the synthesis of stress-induced proteins, the adjustment of cell membrane fatty acid composition, and accumulation of compatible substances [11]. The accumulation of disaccharides (e.g., trehalose, lactose, and sucrose) is a common mechanism underlying the spontaneous response to environmental stress in these organisms. Therefore, the present study focused on disaccharides, which have been widely studied for their anti-freeze-drying effects, specifically the accumulation of disaccharides in cells and the metabolism of disaccharides in the bacteria.

A high-density culture is a fermentation method in which bacteria are cultivated using certain culture techniques and devices to significantly increase the bacterial density compared with conventional culture. This culture technique can thus increase the specific productivity of the target product, ultimately producing more bacteria at a lower cost. Under high-density culture conditions, a hypertonic but less inhibitory environment can be created via constant alkaline supplementation to control the pH of the medium and increase the density of the organisms [12,13,14]. It is valuable to examine whether this high-efficiency culture mode can improve the freeze-drying survival of bacteria.

In this study, we focused on the effects of different sugar additions during bacterial growth on their freeze-drying resistance rather than as freeze-drying protectants and revealed the possible mechanisms. We investigated the effect of the addition of trehalose and lactose, both as a carbon source culture and as an additive, on the freeze-drying survival of *Lactobacillus* in high-density culture. The ability of the bacterium to metabolize the sugar, as well as a culture under hyper-versus hypotonic conditions, was used as a variable factor. Extracellular polysaccharides, cell membrane stability, and intracellular compatible solute accumulation were selected as study targets. The present study aimed to elucidate the mechanisms of the anti-freeze-drying effects of sugars. 

## 2. Materials and Methods

### 2.1. Bacterial Strains, Medium, and Growth Conditions

*Lactobacillus fermentum* FXJCJ6-1, *Lactobacillus reuteri* CCFM1040, and *Lactobacillus brevis* 173-1-2 were obtained from the Culture Collection of Food Microbiology (CCFM) at Jiangnan University (Wuxi, China).

The strains were cultured on MRS plates, and the plates were incubated upside down in a constant temperature incubator at 37 °C for 24–36 h. A single colony was selected from the growing colonies and incubated in MRS liquid medium at 37 °C for 12 h. The seed solution was added to fresh the MRS liquid medium with a 4% inoculum and incubated at 37 °C for 12 h. The activated seed solution was obtained after two to three repetitions.

### 2.2. High-Density Culture of Strains

Based on our preliminary studies, the medium compositions for the strains were designed as follows. The medium for *L. fermentum* FXJCJ6-1 at a constant pH (6.0) batch culture contained 40 g/L yeast extract, 108 g/L glucose, 0.175 g/L MgSO_4_, 0.175 g/L MnSO_4_, and 1 mL/L Tween 80. The medium for *L. reuteri* CCFM1040 at a constant pH (5.0) contained 40 g/L yeast extract, 112 g/L glucose, 0.175 g/L MgSO_4_, 0.175 g/L MnSO_4_, and 1 mL/L Tween 80. The medium for *L. brevis* 173-1-2 at a constant pH (5.0) contained 40 g/L yeast extract, 108 g/L glucose, 0.125 g/L MgSO_4_, 0.125 g/L MnSO_4_, and 1 mL/L Tween 80. 

For the culture mediums of the three *Lactobacillus* strains in different carbon sources, glucose was replaced with lactose, sucrose, or trehalose according to the glucose content in the corresponding basal medium for each strain.

### 2.3. Cell Preparation and Freeze-Drying

#### 2.3.1. Cell Preparation

The fermentation broth was collected, and the supernatant was removed by centrifugation at 8000× *g* and 4 °C for 20 min. The cells were mixed thoroughly with the protectant solution at a mass ratio of 1:1. Next, 1 mL of the mixture was pipetted into an 8 mL freeze-drying glass bottle (id = 22 mm) for freeze-drying. The protective agent solution consisted of oligoisomaltose 75 g/L and collagen 25 g/L.

#### 2.3.2. Freeze-Drying

Freeze drying was carried out using a Telstar freeze dryer (Telstar Cryodos, Terrassa, Spain). The freeze-drying process was set to the following. The temperature of the laminate was controlled to drop to −50 °C from room temperature within 1 h and to maintain this temperature for 4 h. Then, the vacuum degree was controlled, and the temperature of the freeze dryer shelf was adjusted to −30 °C within 1.3 h, which was maintained under a 0.2 mbar vacuum degree for 30 h. Lastly, the temperature of the freeze dryer shelf was controlled for 1 h, raised to 25 °C, and maintained for 20 h under the condition of a 20 μbar vacuum.

### 2.4. Determination of Freeze-Drying Survival Rate

The number of viable bacteria (CFU/mL) in each sample before freeze-drying was determined. Next, sterile saline was added to the freeze-dried powder of the same mass before freeze-drying. Finally, the number of viable bacteria (CFU/mL) in the samples after freeze-drying was determined using the plate colony counting method to calculate the freeze-drying survival rate, expressed as:

$$\mathrm{Freeze}-\mathrm{drying}\;\mathrm{survival}\;\mathrm{rate}\;(\%)=x=\frac{\mathrm{NA}}{\mathrm{NB}}\times 100\;$$

NA—number of live bacteria in the sample before freeze-drying; NB—number of live bacteria in the sample after freeze-drying.

### 2.5. Scanning Electron Microscopy

The fermentation broth (1 mL) was centrifuged at 8000× *g* for 10 min. The supernatant was removed, and the precipitate was washed three times with an isotonic phosphate-buffered saline (PBS) solution to obtain the cells to be examined. Next, 0.75 mL of glutaraldehyde fixative for electron microscopy was added to the centrifuge tube, and the tube was left for 2 h at 4 °C. After centrifugation to remove the supernatant, the samples were rinsed thrice with a PBS solution for 30 min. The samples were then precipitated with ethanol and dehydrated with a 1:1 (*v/v*) mixture of anhydrous ethanol, tert-butanol, and pure tert-butanol solution once for 15 min each. Finally, the samples were dried using a vacuum concentrator. For scanning electron microscopy, the sample was attached using conductive tape to the microscope stage. The surface of the sample was plated with a gold film with a thickness of 100–150 Å. The appropriate magnification and field of view were then selected to observe the morphology of the bacterium.

### 2.6. Intracellular Contents Determination

#### 2.6.1. Extraction of Intracellular Contents

The fermentation broth was centrifuged at 8000× *g* for 15 min. The supernatant was removed, and the sample was washed thrice with an isotonic PBS solution. The extractant (acetonitrile: methanol: water = 2:2:1) was added to the sample and shaken well to suspend the slime in the extractant. The mixture was repeatedly freeze–thawed in liquid nitrogen three times to break the bacterium. The metabolites were dissolved, and the extract was collected. The extraction procedure was repeated. The metabolites were mixed twice and transferred to a centrifuge tube. Finally, the sample was blown dry using a vacuum concentrator and then stored at −80 °C.

#### 2.6.2. Measurement of Intracellular Sugars

The chromatographic conditions for the detection of sugars were as follows. Waters-600 HPLC was used with a Sugarpak I column at a column temperature of 85 °C, using the evaporative light scattering detection method. The mobile phase was ultrapure water, and the flow rate was 0.4 mL/min.

### 2.7. Extracellular Polysaccharide Determination

#### 2.7.1. Extraction of Extracellular Polysaccharides

The bacterial solution was collected, sonicated at 25 °C for 10 min (500 w), and centrifuged at 10,000× *g* for 15 min. Next, the supernatant was removed, and 5% Trichloroacetic acid solution (80%, *v/v*) was added to precipitate the protein. The mixture was stored at 4 °C for 30 min and then centrifuged at 10,000× *g* for 15 min. The supernatant was removed, and anhydrous ethanol was added to the supernatant (supernatant: anhydrous ethanol = 1:9, *v/v*) for alcoholic sedimentation at 4 °C overnight. After centrifugation at 10,000× *g* for 15 min, a flocculent precipitate was obtained, collected, dissolved in an equal volume of ultrapure water, and packed into a dialysis bag (8000 D). The water was changed once at 8 h. After 48 h of ultrapure water dialysis, the precipitate was removed and lyophilized to obtain extracellular polysaccharides.

#### 2.7.2. Determination of Extracellular Polysaccharides

Anthrone (0.2 g) was dissolved in 100 mL of 80% concentrated sulfuric acid. The above polysaccharide precipitate was dissolved in ultrapure water and diluted with water to 1 mL. Next, 4 mL of sulfuric acid-anthrone solution was added. The mixture was then removed from the boiling water bath for 10 min and cooled to room temperature. The OD_620_ was determined, and the extracellular polysaccharide content was calculated according to the standard curve.

### 2.8. Statistical Analysis

All experiments were repeated three times, and the data are presented as the mean ± standard deviation. Experimental data were plotted using Origin 9.1, and one-way ANOVA (Duncan’s test) was performed using SPSS (version 25.0; SPSS Inc., Chicago, IL, USA). Statistical significance was set at *p* < 0.05.

## 3. Results

### 3.1. Determination of the Freeze-Drying Survival of LAB with Different Sugars as the Sole Carbon Source 

Three *Lactobacillus* strains were cultured at high density using different sugars as the carbon source and then were lyophilized to determine the number of viable bacteria and the freeze-drying survival rate (Table 1). The freeze-drying survival rate is the most important target, but at the same time, a sufficient number of viable bacteria should be guaranteed. As shown in Table 1, *L. fermentum* FXJCJ6-1 was able to utilize glucose, lactose, and sucrose, and its freeze-dried bacterial count and freeze-drying survival rate were significantly higher after culture with lactose than after culture with glucose. Moreover, *L. reuteri* CCFM1040 was able to utilize glucose and lactose, and its freeze-drying count and survival rate were also higher with lactose than with glucose. By contrast, *L. brevis* 173-1-2 was only able to utilize glucose. For *L. fermentum* FXJCJ6-1 and *L. reuteri* CCFM1040, the freeze-dried bacterial counts and freeze-drying survival rates were both higher after incubation with lactose than glucose or sucrose.

### 3.2. Effects of Mixed Sugars on the Freeze-Drying Survival Rate of LAB

**Figure 1:**
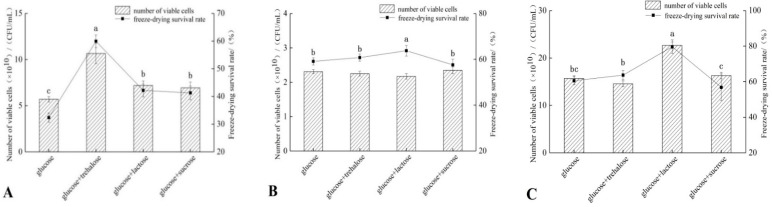
The freeze-drying survival rate and number of viable bacteria of LAB with adding different carbohydrates (2 g/L). (**A**) *L. fermentum* FXJCJ6-1; (**B**) *L. reuteri* CCFM1040; (**C**) *L. brevis* 173-1-2. Different letters indicate significant differences (*p* < 0.05).

Three *Lactobacillus* strains were cultured at high density with glucose as the base carbon source. Trehalose, lactose, and sucrose were then added to the medium at the concentration of 2 g/L, followed by freeze-drying to determine the number of viable bacteria and the freeze-drying survival rate (Figure 1). 

As shown in Figure 1, the sugar addition improved the freeze-drying survival rate by varying degrees. These findings indicate that sugar addition before culture exerts a great anti-freeze-drying effect. 

The addition of trehalose significantly increased the freeze-drying survival of *L. fermentum* FXJCJ6-1 by (27.53 ± 1.36)% compared to the glucose group (*p* < 0.05). Moreover, the addition of lactose significantly increased the freeze-drying survival of *L. reuteri* CCFM1040 and *L. brevis* 173-1-2 compared to the glucose group (*p* < 0.05). As shown in Table 1, *L. fermentum* FXJCJ6-1 could not utilize trehalose, and *L. brevis* 173-1-2 could not utilize lactose. Therefore, the addition of non-metabolizable sugars may have beneficial effects on freeze-drying resistance. Thus, we will focus on *L. fermentum* FXJCJ6-1 and *L. brevis* 173-1-2 to study their freeze-drying resistance with the addition of trehalose or lactose, respectively.

### 3.3. Effects of Trehalose Addition on the Freeze-Drying Resistance of L. fermentum fXJCJ6-1 in Different Conditions

**Figure 2:**
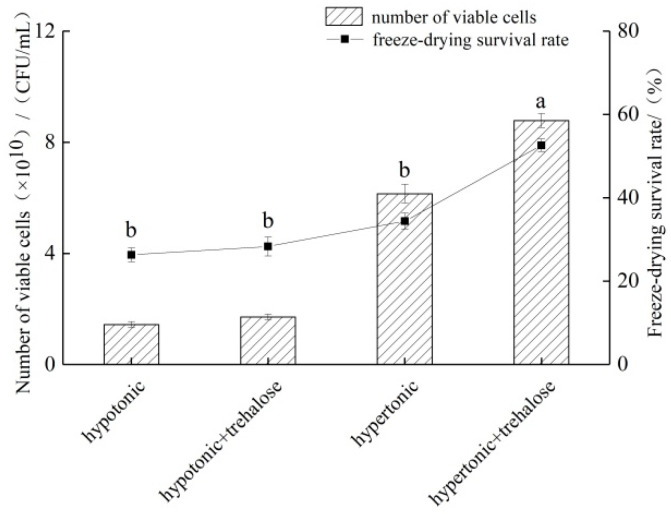
The effect of adding trehalose in hypertonic and hypotonic conditions on the survival rate and viable bacteria of *L. fermentum* FXJCJ6-1. Different letters indicate significant differences (*p* < 0.05).

The above results show that the addition of trehalose greatly enhanced the freeze-drying survival of *L. fermentum* FXJCJ6-1, but this strain was unable to utilize trehalose as a carbon source. Combined with the fact that bacteria have been found to take up compatible solutes from outside to counteract adverse stress in a hypertonic environment, several studies have shown that the accumulation of carbohydrates in their cells could help bacteria resist desiccation stress [15]. High intracellular trehalose concentrations have also been observed to enhance stability in yeast, mammalian, and plant cells under freezing or desiccation stresses [15,16]. It has also been shown that some organisms actively accumulate trehalose to resist desiccation stress [17]. We speculated that the hypertonic culture environment may have allowed the bacterium to initiate its own resistance mechanism using trehalose. Therefore, we compared the effect of trehalose addition under hypertonic and hypotonic conditions on the freeze-drying survival of the strains. As shown in Figure 2, the freeze-drying survival rate of *L. fermentum* FXJCJ6-1 when cultured in a hypertonic environment was significantly higher than that of a hypotonic environment (*p* < 0.05). Moreover, only the addition of trehalose in the hypertonic environment could significantly increase the freeze-drying survival rate of the strain (*p* < 0.05). Therefore, it was speculated that *L. fermentum* FXJCJ6-1 intracellularly accumulated trehalose as a compatible solute in the hypertonic environment to improve resistance to adversity.

To test the conjecture that *L. fermentum* FXJCJ6-1 accumulates trehalose intracellularly to improve its resistance during freeze-drying, we examined the intracellular and extracellular trehalose content of *L. fermentum* FXJCJ6-1 when grown under hypertonic and hypotonic environments (Table S1). The results showed that there was no significant decrease in trehalose content in the supernatant of *L. fermentum* FXJCJ6-1 grown under hypotonic conditions. However, trehalose content in the supernatant of *L. fermentum* FXJCJ6-1 decreased by 5.5% under hypertonic conditions. Thus, the intracellular trehalose concentration was further examined. Our findings showed the presence of intracellular trehalose but did not provide an accurate quantity. This may be due to the low amount of trehalose transported by the strain or the loss of trehalose during treatment, which needs to be examined more accurately in the future. Because *L. fermentum* FXJCJ6-1 has the possibility of transporting trehalose into the cell, we annotated the gene sketch of *L. fermentum* FXJCJ6-1 and selected sugar transporter-related genes. After comparing the amino acid sequence with that of treB, we found that PTS-II-sucr in *L. fermentum* FXJCJ6-1 was similar to the amino acid sequence of treB (Figure S1) (Max Score: 235; Query Cover: 98%; E value: 2 × 10^−74^; Per. Ident: 30.38%). Therefore, we speculate that this gene is similar in function to treB and capable of transporting trehalose into the cytosol. In contrast, no genes related to trehalose transport were identified in *L. reuteri*.

### 3.4. Effects of Lactose Addition on the Freeze-Drying Resistance of L. brevis 173-1-2 in Different Conditions

**Figure 3:**
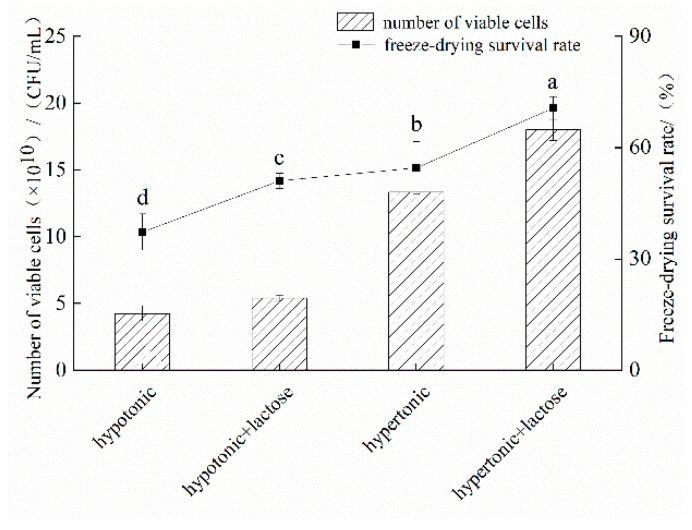
The effect of adding lactose in hypertonic and hypotonic conditions on the survival rate and viable bacteria of *L. brevis* 173-1-2. Different letters indicate significant differences (*p* < 0.05).

As shown in Figure 3, the freeze-drying survival of *L. brevis* 173-1-2 was significantly increased by the addition of lactose under both hypo- and hypertonic conditions. In conjunction with the results in Figure 2, lactose was translocated into the cell of *L. brevis* 173-1-2. Thus, the intracellular lactose content of *L. brevis* 173-1-2 when grown under hypo- and hypotonic conditions was examined using HPLC, and the results indicate that neither hypo- nor hypotonic conditions promoted the intracellular lactose content of *L. brevis* 173-1-2 (Table S2). Therefore, the hypothesis that *L. brevis* 173-1-2 accumulates in lactose intracellularly to enhance its resistance to hypo- and hypertonic stresses is not valid. As *L. brevis* 173-1-2 was unable to metabolize lactose and hypertonic stress did not induce the translocation of lactose into the cells, we speculated that lactose, if added as a small molecular semi-permeable solute, could permeate the bacterial cell wall without entering the cell membrane and protect membrane stability in this space, thereby exerting an anti-freeze-drying effect [18] and preventing cell damage due to ice crystal formation [19].

### 3.5. Potential Mechanisms for the Increase in Freeze-Drying Survival of L. fermentum FXJCJ6-1 and L. reuteri CCFM1040 by Lactose Addition

As shown in Table 1, *L. fermentum* FXJCJ6-1 and *L. reuteri* CCFM1040 were able to metabolize lactose, and their lyophilized survival rate significantly increased after incubation in a lactose medium. After testing the sugar transport theory, we examined the intracellular contents of *L. fermentum* FXJCJ6-1 and *L. reuteri* CCFM1040 under hypertonic and hypotonic conditions. The results revealed no intracellular accumulation of lactose in *L. fermentum* FXJCJ6-1 and *L. reuteri* CCFM1040. This negates the possibility of *L. fermentum* FXJCJ6-1 and *L. reuteri* CCFM1040 accumulating lactose intracellularly to enhance their resistance to freeze-drying.

Studies have shown that changes in the substrate of a culture medium can lead to changes in the morphology of bacteria after growth. Some of these changes (e.g., transformation to a spherical shape or change in the membrane structure) can lead to increased resistance to freeze-drying [20]. Therefore, we tested this hypothesis by comparing the strains cultured with lactose with those cultured in the control (MRS) medium (Figure S2). Our results showed no significant morphological change in *L. fermentum* FXJCJ6-1 and *L. reuteri* CCFM1040 after incubation with and without lactose, thus ruling out the possibility that the lactose culture affects the morphology and size of lactic acid bacteria.

**Figure 4:**
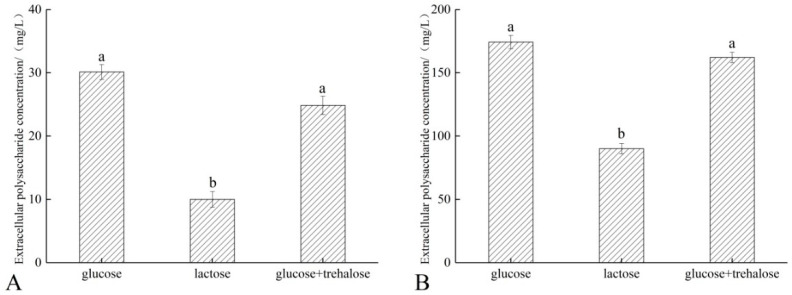
Extracellular polysaccharide production of *L. fermentum* FXJCJ6-1 (**A**) and *L. reuteri* CCFM1040 (**B**) with different sugars. Different letters indicate significant differences (*p* < 0.05).

Previous studies have shown that changes in culture conditions can lead to changes in extracellular polysaccharide production by bacteria [10]. As shown in Figure 4, the production of extracellular polysaccharides by *L. reuteri* CCFM1040 and *L. fermentum* FXJCJ6-1 was significantly reduced in the medium with lactose as the sole carbon source (*p* < 0.05). Extracellular polysaccharides contribute to the resistance of an organism to stress [21]. However, this effect may diminish when the amount of extracellular polysaccharides reaches a certain level [22]. When the bacteria produced only a small amount of extracellular polysaccharides, the small molecules of the freeze-drying protectant were easily bound to the polar groups on the cell membrane through the cell wall and were less likely to be affected by extracellular polysaccharides [23]. As a result, the effective protective layer formed by hydrogen bonds between the cell wall and the cell membrane was not destroyed, which helped improve the survival rate of the strains.

## 4. Discussion

Sugars are an important component of microbial media, not only as a carbon source to provide nutrients for cell proliferation but also as they influence the stress resistance of microbes. Different sugars affect the freeze-drying resistance of *Lactobacillus*, mainly in the following aspects: first, the addition of different sugars to the culture media will affect the morphology and size of bacteria; second, in a hypertonic environment, some strains resistant to high osmotic pressure are able to transfer compatible solutes, such as trehalose and sucrose, into the cell, thereby regulating the osmotic pressure balance inside and outside the cell and improving the resistance of the strain itself; third, sugars in the medium can affect the extracellular polysaccharide production of lactic acid bacteria. The reduction in polysaccharides increases the efficacy of the protective agent on the bacterium. In this study, we investigated whether the metabolism of trehalose and lactose by *Lactobacillus* strains influenced their freeze-drying survival rate after incubation under hypertonic conditions. Our hypotheses were as follows: (1) strains that could not metabolize trehalose and lactose had increased freeze-drying survival rates after the addition of trehalose and lactose, and they could intracellularly accumulate in trehalose and lactose under hypertonic conditions, leading to an increase in their freeze-drying survival rate; (2) strains that could metabolize lactose had increased freeze-drying survival rates after incubation with lactose, either because of changes in morphology and extracellular polysaccharide production during lactose fermentation or via the intracellular accumulation of lactose, leading to increased freeze-drying survival rates.

We also investigated the freeze-drying resistance mechanism and found that cultures under hypertonic conditions induced *L. fermentum* FXJCJ6-1 to intracellularly accumulate trehalose to resist freeze-drying stress. The freeze-drying survival of *L. fermentum* FXJCJ6-1 increased by (27.53 ± 1.36)% after trehalose was added to the medium. This finding is similar to those of other studies [24]. Song et al. found that high sugar concentrations in the medium promoted the accumulation of trehalose [25]. MD Angelis et al. found that the enzymes involved in the synthesis of compatible solutes by *Lactobacillus* were inhibited in a hypertonic environment and that cells translocated compatible solutes from the environment to resist environmental stresses [26]. When osmotic pressure decreases, cells release intracellular compatible solutes through a specific efflux system to restore intra- and extracellular osmotic equilibrium; when the osmotic pressure increases, cells transport water to the extracellular compartment and accumulates compatible solutes in the intracellular compartment. Therefore, the accumulated level of compatible solutes, such as trehalose, is determined by the osmotic pressure of the environment to which a bacterium is exposed. In addition, Hounsa et al. found that under high osmolarity conditions, brewer’s yeast accumulate intracellular trehalose during the stable phase, leading to a significantly higher freeze-drying survival in the stable phase than in the log phase [27]. Diniz-mendes et al. found that yeast cells containing 4-5% trehalose could protect themselves from freezing damage; the mechanism of freezing tolerance in most freeze-tolerant yeasts involves the maintenance of high intracellular trehalose levels during fermentation [28]. Consistent with a previous finding that the transporter protein treB in *L. acidophilus* is a trehalose-transporting protein [24], we found that *L. fermentum* FXJCJ6-1 also has the ability to transport trehalose. 

When a trehalose-transporting protein is present in a bacterium, hypertonic conditions may induce the transport of trehalose into the cytosol, thereby increasing its resistance to stress. Therefore, further investigation of the mechanisms by which environmental conditions affect trehalose transport can be carried out using this gene. Moreover, other strains with trehalose transport systems can be predicted for the more effective examination of the anti-freeze-drying effect of trehalose.

In addition, we found a significant reduction in the extracellular polysaccharide production for *L. fermentum* FXJCJ6-1 and *L. reuteri* CCFM1040 grown in a lactose culture medium, whereas their freeze-drying survival increased by (24.92 ± 1.74)% and (15.68 ± 2.61)%, respectively. Several studies have found that extracellular polysaccharides are key substances for improving the freeze-drying survival of *Lactobacillus* [21,29,30]. Extracellular polysaccharides are firmly bound to the cell surface and protect cells against adverse conditions, such as high temperatures, high osmolarity, and low acidity [31]. Joseph et al. isolated exopolysaccharides from *Colwellia psychrerythraea,* incubated the bacterium with the exopolysaccharides as a protective agent, and lyophilized the culture [21]. Compared with other protective agents, exopolysaccharides led to a higher survival rate of the bacterial cells. Moreover, the properties of the extracellular polysaccharide may be similar to those of polymers with a glassy state and, therefore, have a protective effect on the bacterium at low temperatures [32]. However, this does not mean that higher extracellular polysaccharide levels are favorable. Our study demonstrated that the reduction in extracellular polysaccharides was beneficial for inducing the freeze-drying resistance of the strains and had a positive effect on the freeze-drying survival rate of the strains.

## 5. Conclusions

Overall, we found significant differences between the efficacies of trehalose and lactose in improving the freeze-drying survival of *Lactobacillus* stains. The addition of trehalose and lactose during bacterial growth may significantly increase their freeze-drying survival. Hypertonic conditions were beneficial for the transport of trehalose into the bacterial cell, thereby improving the freeze-drying resistance of *L. fermentum* FXJCJ6-1. Lactose as a carbon source also significantly improved the freeze-drying survival of *L. fermentum* FXJCJ6-1 (A) and *L. reuteri* CCFM1040. One possible reason may be the decrease in the production of extracellular polysaccharides after incubation with lactose, which was conducive to the protective effect of the freeze-drying protectant. Overall, our results provide an alternative strategy to improve the freeze-drying survival of LAB strains, as well as theoretical support for the addition of suitable sugars in the culture process.

## Figures and Tables

**Table 1:** The number of viable bacteria and freeze-drying survival rate of LAB in different sugars.

	*L. fermentum* FXJCJ6-1		*L. reuteri* CCFM1040		*L. brevis* 173-1-2	
	Number of Active Bacteria (×10^10^)/ (CFU/mL)	Freeze-Drying Survival Rate/(%)	Number of Active Bacteria (×10^10^)/(CFU/mL)	Freeze-Drying Survival Rate/(%)	Number of Active Bacteria (×10^10^)/ (CFU/mL)	Freeze-Drying Survival Rate/(%)
Glucose	5.7 ± 0.29	32.39 ± 1.67 ^a^	2.31 ± 0.06	59.1 ± 1.57 ^a^	15.68 ± 0.59	60.52 ± 2.26
Trehalose	-	-	-	-	-	-
Lactose	10.88 ± 0.95	57.31 ± 1.74 ^c^	3.47 ± 0.14	66.78 ± 2.61 ^b^	-	-
Sucrose	7.05 ± 0.29	38.21 ± 1.56 ^b^	-	-	-	-

Note: ‘-‘ means no growth on this sugar. Different letters indicate significant differences (*p* < 0.05).

## Data Availability

Not applicable.

## Supplementary Materials

The following supporting information can be downloaded at: https://www.mdpi.com/article/10.3390/microorganisms11010048/s1.
