# Supplementary material for: Effect of Trehalose and Lactose Treatments on the Freeze-Drying Resistance of Lactic Acid Bacteria in High-Density Culture
Source: Microorganisms. 2022 Dec 23;11(1):48. doi: 10.3390/microorganisms11010048 (PMC9866448; doi:10.3390/microorganisms11010048)
Supplement: Supplementary file 1 [file microorganisms-11-00048-s001.zip › microorganisms-2004834-supplementary.pdf]

Supplementary Information

Supplemental Tables

Table S1. The intracellular and extracellular trehalose content of *L. fermentum* FXJCJ6-1 grown under hypo- and hypertonic conditions.

Conditions	Extracellular trehalose content (g/L)	Intracellular trehalose content (g/L)
hypotonic	2.00 ± 0.01	-
hypertonic	1.89 ± 0.04	-

Note: '-' means not detected.

Table S2. The intracellular lactose content of *L. brevis* 173-1-2 grown under hypo- and hypertonic conditions.

Conditions	Intracellular lactose content（g/L）
hypotonic	-
hypertonic	-

Note: '-' means not detected.

Supplemental Figures

```
Query  1    MMSKINQTDIDRLIELVGGRGNIATVSHCITRLRFVLNQPANARPKEIEQLPMVKGCFTN  60
            M  K   TD+ + I    G  NI   +HC TRLR VL        K ++    VKG F
Sbjct  1    MDHKQVATDVIKAI----GADNIVAAAHCATRLRLVLKDDEAIDQKALDNNDGVKGTFKT  56

Query  61   AGQFQVVIGT-NVGDYYQALIASTGQAQVDKEQVKKAARHNMKWHE--QLISHFAVIFFP  117
             GQ+QV+IG  +V + Y  LI  TG  +V  E +K+    N K++      I   + IF P
Sbjct  57   NGQYQVIIGPGDVNNVYDELIKQTGLKEVSTEDLKQIVAKNQKFNPVMAFIKLLSDIFVP  116

Query  118  LLPALISGGLILGFRNVIGDLPMSNGQTLAQMYPSLQTIYDFLWLIGEAIFFYLPVGICW  177
            ++PAL++GGL++   NV+     +    +++ QM+P+++ +    + ++  A + +LP+ +
Sbjct  117  IIPALVAGGLLMALDNVLVSQGLFGPKSVVQMFPAIKGLEGMVNVMSAAPYVFLPILVGV  176

Query  178  SAVKKMGGTPILGIVLGVTLVSPQLMNAYLLGQQLPEVWDFGMFSIAKVGYQAQVIPALL  237
            S  K+ G    LG V+G+ + +P L             W    F++     Y  QVIPAL
Sbjct  177  SGAKRFGANQFLGAVVGMMMTAPVLAEG--------GAWHLFGFTVNIQSYTGQVIPALA  228

Query  238  AGLALGVIETRLKRIVPDYLYLVVVPVCSLILAVFLAHALIGPFGRMIGDGVAFAVRHLM  297
            A   L + E     + +P  +     P+ S+IL  F+    ++GP  + + D +   +  L
Sbjct  229  AVWILSIFEKWFHKKLPSAVDFTFTPLLSVILTGFITFIVVGPVMKELSDLITNGIVWLY  288

Query  298  TGSFAPIGAALFGFLYAPLVITGVHQTTLAIDLQMIQSMG-----GTPVWPLIALSNIAQ  352
            + +   +G  +FG +Y+P+V+TG+HQ+  AI+ Q++ +       G  ++ + +++N+AQ
Sbjct  289  S-TLGFVGTGIFGAIYSPIVLTGLHQSFPAIETQLVTAYKSGTGYGDFIFVVASMANVAQ  347

Query  353  GSAVIGIIISSRKHNEREISVPAAISAWLGVTEPAMYGINLKYRFPMLCAMIGSGLAGLL  412
            G+A    +    ++     + +S  AA+SA LG+TEPA++G+NLK +FP  CA+IGS  A  L
Sbjct  348  GAATTAVYFLTKNEKMKGLSSSAAVSALLGITEPALFGVNLKLKFPFFCALIGSATAATL  407

Query  413  CGLNGVMANGIGVGGLPGILSIQPSYWQVFALAMAIAIIIPIVLTSFIYQRKYR  466
             GL  V+A  +G  G  G LSI       + L   ++ ++   +T F+Y + +
Sbjct  408  AGLFHVIAVSMGSAGFIGFLSIGAKSIPFYLLCELVSFVVAFTIT-FLYGKTHN  460
```

Figure S1. Results of amino acid sequence comparison between PTS-II-sucr in *L. fermentum* FXJCJ6-1 and treB.

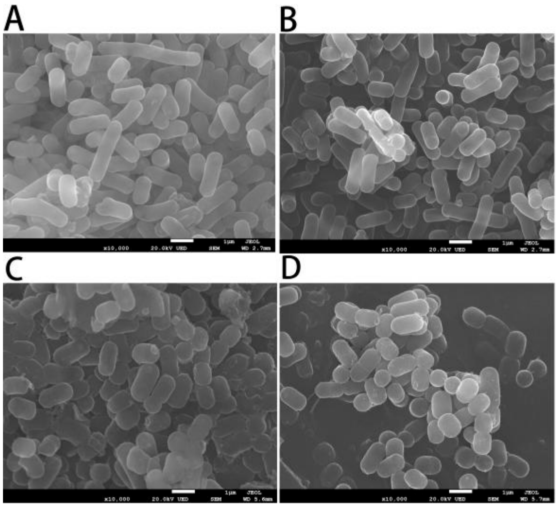

Figure S2. Scanning electron micrographs of the morphology of heterofermentive *Lactobacillus* fermented with different carbohydrates. (A, B-) The bacterial morphology of *L. fermentum* FXJCJ6-1 fermented with glucose and lactose; (C, D)-Bacterial morphology of *L. reuteri* CCFM1040 fermented with glucose and lactose.
